# Rosuvastatin Decreases the Formation of Neointima by Increasing Apo J, Reducing Restenosis after Balloon Injury in Rats

**DOI:** 10.5935/abc.20180204

**Published:** 2018-10

**Authors:** Paulo Magno Martins Dourado

**Affiliations:** Faculdade de Medicina da Universidade de São Paulo - Instituto do Coração (InCor) - Laboratório de Hipertensão Experimental, São Paulo, SP - Brazil

**Keywords:** Clusterin/genetic, Rats, Carotid Arteries, Angioplasty, Percutaneous Coronary Intervention/trends, Coronary Restenosis

The study by Yang et al.^1^ brings new light on the action of Apolipoprotein (Apo) J, also called clusterin (CLU), a heterodimeric glycoprotein consisting of α and β subunits linked by disulfide bond.^2^^,^^3^ The coding gene of Apo J is located on chromosome 8p21-p12, encoding two main isoforms, including secreted (sCLU) and nuclear (nCLU)^4^ on restenosis following balloon percutaneous transluminal carotid angioplasty (PTCA) in an experimental model in rats; this was published in this edition of the Brazilian Archives of Cardiology, because there is a controversy in the literature whether Apo J, which is elevated in atherosclerosis and post-angioplasty, plays a protective or promoting role for restenosis. Apo J is involved in several important pathological processes in the transport of lipids, and in the differentiation of vascular smooth muscle cells (VSMC), including cell death by apoptosis, cell cycle regulation, cell adhesion, tissue remodeling, regulation of the immune system and oxidative stress, playing a role in the development of clinical atherosclerosis.^5^^,^^6^ In the process of attenuating atherosclerosis, Apo J can promote the export of cholesterol and phospholipids from foam cells of macrophages,^7^ and show cytoprotective and anti-inflammatory actions interacting with many known inflammatory proteins that may act in the initial phase of clinical cardiovascular events, and may play an important role in mediating atherosclerotic disease, such as C-reactive protein, paraoxonase and leptin.^8^ There are studies reporting that Apo J can stimulate the proliferation and migration of VSMC, and promote restenosis,^9^^,^^10^ and there have been studies showing that overexpression of CLUs can inhibit migration and proliferation of VSMC, and inhibit cellular apoptosis.^11^ In view of these controversial results, the authors sought to elucidate the role of Apo J in neointimal hyperplasia, using the rat carotid artery in vivo, with or without rosuvastatin.

The results of the study published here suggest that rosuvastatin can inhibit intimal hyperplasia due to the high expression of Apo J in the active proliferation and migration phase of VSMC, after balloon injury in rats. In the present study, the authors evidenced an increase in the intimal/media (I/M) area rate after the balloon injury that reached the maximum value in the fourth week in the model group; in addition, I/M was increased at week 2, and such increase ceased after the administration of rosuvastatin. These results suggest that rosuvastatin can significantly reduce the degree of intimal hyperplasia in the balloon-injured carotid arteries in rats. The levels of Messenger Ribonucleic Acid (mRNA) and Apo J were increased in the carotid arteries in the group using rosuvastatin, when compared to the model group, reaching the maximum in the second week, earlier than in the model group, suggesting that rosuvastatin can inhibit intimal hyperplasia by increasing Apo J after balloon injury in rats. Therefore, Apo J has been identified as having a central role in the migration, adhesion and vascular proliferation process, and that it can contribute significantly to restenosis after vascular injury.

The results of this study showed that Apo J can be an acute phase reagent after balloon injury in the carotid arteries of rats; therefore, it plays a favorable role, decreasing the development of restenosis, which in spite of all existing interventions remains as a challenge to be overcome. Rosuvastatin, a potent inhibitor of the HMG-CoA (hydroxymethylglutaryl-CoA) reductase enzyme, seems to reduce neointimal thickening after vascular endothelial injury in rats.

This study opens new perspectives by highlighting possible mechanisms involved in the genesis of restenosis after percutaneous interventions, opening the way for clinical studies researching the action of Apo J as a new predictor and a therapeutic target for the protection of the vessel after PTCA.
